# The effect of feeding with different protein levels on internal organ weight and gene expression of MEF2A and ATF3 in crossbred local chicken using RT-PCR

**DOI:** 10.1186/s43141-023-00533-6

**Published:** 2023-08-11

**Authors:** Alfan Kurniawan, Muhammad Halim Natsir, Suyadi Suyadi, Osfar Sjofjan, Yuli Frita Nuningtyas, Ari Ardiantoro, Ahmad Furqon, Suci Puji Lestari

**Affiliations:** 1 Department of Nutrition and Animal Feed, Faculty of Animal Science, Universitas Brawijaya, Malang, East Java 65145 Indonesia; 2 Faculty of Animal Science, Universitas Brawijaya, Malang, East Java 65145 Indonesia; 3 Research Center for Applied Zoology, National Research and Innovation Agency Republic of Indonesia, Jakarta, 16911 Indonesia

**Keywords:** ATF3, Crossbred local chicken, Gene expression, Internal organs, MEF2A, RT-PCR

## Abstract

**Background:**

Myogenic enhancer transcription factor 2A (MEF2A) is a transcription factor known for its role in controlling skeletal muscle regeneration and metabolic processes, while activating transcription factor 3 (ATF3) is a stress-induced transcription factor that plays a role in modulating metabolic processes, immunity, and oncogenesis. Environmental factors, such as dietary protein, can influence gene expression levels. Insufficient protein intake can negatively affect the metabolic performance of internal organs, leading to the abnormal weight of internal organs. A total of 192 non-sexing crossbred local chickens day-old-chick (DOC) with a completely randomized design (CRD) method of 3 treatments and 8 replicates. Real-time polymerase chain reaction (RT-PCR) is used to measure the gene expression levels. This study aimed to determine the effect of feeding with various protein levels on internal organ weight and gene expression of MEF2A and ATF3 in crossbred local chickens.

**Result:**

The analysis of treatment revealed that the results were not significantly different (*P* > 0.05) on gizzard weight and spleen weight. However, it shows a significantly different result (*P* < 0.05) on heart weight and a highly significantly different result (*P* < 0.01) on pancreas weight. These findings suggest that protein levels in the diet had a significant impact on heart and pancreas weights. In terms of gene expression, the increased utilization of protein did not result in an elevation of MEF2A gene expression in both muscle tissue and liver tissue. Specifically, in muscle tissue, MEF2A gene expression was highly expressed at 18% protein feed for the starter phase and 16% for the finisher phase. Conversely, in liver tissue, MEF2A gene expression was highly expressed at 22% protein feed for the starter phase and 20% for the finisher phase. Moreover, ATF3 gene expression in muscle tissue exhibited a negative correlation with increasing feed protein levels.

**Conclusion:**

The results indicate that varying protein levels did not lead to abnormal weights in the liver, kidney, heart, and spleen organs. Additionally, the differential gene expression patterns of MEF2A and ATF3 in muscle tissue and liver tissue suggest that these genes respond differently to varying protein-feeding treatments. These findings provide insights into the complex regulatory mechanisms of MEF2A and ATF3 genes in relation to protein levels and organ-specific responses in crossbred local chickens.

## Background

Indonesia is home to various chicken breeds, including local chickens, exotic chickens, and local crossbreds. Local chickens represent indigenous Indonesian breeds, such as Merawang Chicken, Nagrak Chicken, Sentul Chicken, and Gaok Chicken, which are commonly raised by local communities. Exotic chickens, such as Pelung Chicken, Tukung Chicken, and Bekisar Chicken, possess distinctive characteristics in terms of body shape, feathers, posture, color, and voice. Crossbred local chickens are obtained through the mating of male native chickens with female laying hens [1]. These crossbred chickens exhibit faster weight gain compared to local chickens. The primary objective of raising crossbred local chickens is to enhance genetics and productivity, resulting in increased body weight and carcass percentage [2]. Carcass percentage is influenced by factors such as livestock breed, age, sex, feed, abdominal fat weight, and internal organ weight [3]. Metabolic disorders in poultry can occur when the nutrients in the feed do not meet the specific requirements of the animals [4]. Abnormal liver, kidney, heart, and spleen weights are indicators of fat metabolism, carbohydrate metabolism, and protein metabolism disorders [5].

Protein is an essential nutrient in livestock feed, fulfilling basic needs, supporting production, facilitating the growth of new tissues, repairing damaged tissues, and serving as a source of metabolic energy [1]. The composition of feed nutrients, particularly protein provided during the growth phase, plays a crucial role in optimizing organ growth and development [6]. Currently, there is no standardized feed formulation available specifically tailored to the needs of local crossbred chickens. It is important to adjust the protein content in the diet to meet the requirements of poultry, as excessive protein content in the livestock’s body will be excreted as ammonia.

Conversely, low protein content in the feed negatively impacts organ growth performance in chickens [7]. Modulating the protein and energy content of the diet according to the specific needs of the chickens is an effective strategy to enhance productivity and reduce stress levels in broilers [8]. Discrepancies in the nutritional composition received by livestock have a significant impact on gene expression responses [9]. Genetic information in livestock is utilized to support the development of breeding and conservation strategies [10]. Nutrigenomics is a branch of molecular biology in animal nutrition, which focuses on studying the impact of feed nutrition on gene expression and its subsequent effects on animal phenotypes [11]. The gene expression results from cell sensors that detect feed nutrition signals as genetic information consumed by livestock. In this context, MEF2A and ATF3 genes are of particular interest as they play critical roles in metabolism and growth. The MEF2A gene controls skeletal muscle regeneration and metabolic processes, exhibiting high expression levels in muscle, heart, and brain tissues while being lowly expressed in kidney and liver tissues [12]. The ATF3 gene, a stress-induced transcription factor, modulates metabolic processes, immunity, and oncogenesis [13]. Increased stress levels adversely affect growth and health in poultry by compromising immunity and leading to excessive fat accumulation [14]. Gene expression is regulated by cellular sensors that detect feed nutrient signals, with proteins containing amino acids serving as cell signaling molecules, phosphorylating proteins, and acting as gene expression regulators [14]. Real-time polymerase chain reaction (RT-PCR) is employed for gene expression analysis due to its accuracy, efficiency, and rapid transcript analysis [15]. This research aims to investigate the effects of different protein levels in the diet on internal organ weight and gene expression of MEF2A and ATF3 in crossbred local chickens.

## Methods

### Experimental design, birds, and diets

This research employed a randomized complete block design and utilized a total of 192 non-sexing local day-old chicks (DOC) obtained from Berline Farm. The experiment was conducted between September and November 2021 at Berline Farm, located in Maguan Village, Ngajum District, Malang Regency, East Java, Indonesia. The chickens were reared for a duration of 8 weeks and subjected to three different treatments, each with 8 replications. The experimental facility consisted of open houses divided into 24 plots, with each plot measuring 1 m × 1 m × 2 m. The feed formulation included various ingredients such as yellow corn, separator bran, fish meal, soybean meal, corn distillers dried grains with solubles (DDGS), copra meal, broiler concentrate, salt, coconut oil, dl-methionine, and premix. The feed was prepared by self-mixing, with all the ingredients thoroughly blended using a vertical mixer. The chickens had ad libitum access to feed and drinking water provided in the morning and evening. The study differentiated between two growth phases: the starter phase, ranging from 0 to 28 days of age, and the finisher phase, spanning from 29 to 56 days of age.

The treatments in the research were as follows:

- T1) Crude protein 18% for the starter and 16% for the finisher.
- T2) Crude protein 20% for the starter and 18% for the finisher.
- T3) Crude protein 22% for the starter and 20% for the finisher.

### Tissue sampling

Sampling collection was performed by slaughtering the chickens using a knife. The chicken’s neck was precisely cut at the jugular vein and carotid artery. Subsequently, the internal organs, including the kidneys, heart, gizzard, and spleen, were collected as phenotypic data. Muscle tissue samples were obtained from the pectoral muscle (pectoralis major), while liver tissue samples were taken from the interior region, serving as measurements of gene expression. Tissue sampling was conducted promptly and meticulously to minimize contamination and tissue damage during the RNA extraction process. Sterilized tweezers and scissors, treated with 70% alcohol and RNAse Away, were used for tissue collection. The collected samples were placed in 1.5-ml tubes containing a DNA/RNA shield. Each tube was labeled with a sample code using label paper and subsequently transferred to a cool box filled with ice cubes. The samples were immediately stored in a freezer for further analysis. This rigorous sampling procedure ensured the preservation of tissue integrity and minimized the risk of sample degradation, enabling accurate analysis of gene expression.

### Measurement of internal organ weight

Samples of internal organs, including the kidneys, heart, gizzard, and spleen, were carefully cleaned to remove any external debris. Subsequently, the samples were weighed using analytical scales to determine the weight in grams of each internal organ. This procedure allowed for the assessment of organ weight in local crossbred chickens subjected to varying protein levels. The weighing process was conducted meticulously to ensure accurate measurements and minimize errors. The recorded weights of the internal organs provided valuable data for evaluating the effects of different protein levels on organ development and growth in local crossbred chickens.

### RNA isolation and reverse transcription

Muscle tissue and liver tissue samples were isolated from the local crossbred chickens using the Zymo Research Quick-RNA™ Miniprep Plus extraction kit (California) following the manufacturer’s protocol. This extraction kit is known for its efficiency in obtaining high-quality RNA samples. The extracted RNA samples were then assessed for their quantity and purity using a Nanodrop spectrophotometer. The purity of the RNA was determined by measuring the absorbance ratio at 260/280 nm, with values ranging from 1.8 to 2.0 considered indicative of high-quality RNA [16]. To convert the RNA into complementary DNA (cDNA), a reverse transcription PCR (RT-PCR) process was carried out. This process involved the use of a reverse transcriptase enzyme to synthesize cDNA from the RNA template. The reverse transcription reaction mixture included various components, such as the tissue samples, 4 × DNA master mix, gDNA remover, nucleus-free water, and 2 µl 5 × RT master mix II. The resulting cDNA products obtained from the reverse transcription process were stored at − 20 °C for further analysis, specifically for subsequent RT-PCR experiments.

### Genes primer design

The primer sequences for the MEF2A gene, ATF3 gene, and the housekeeping gene GAPDH were designed based on sequences obtained from the GenBank database (https://www.ncbi.nlm.nih.gov). The primer design aimed to specifically target the desired DNA fragments for amplification. The primer sequences were analyzed using the Molecular Evolutionary Genetics Analysis (MEGA) software, version 11. This software is commonly used for various genetic analysis tasks, including primer design and sequence alignment. In eukaryotic cells, mRNA sequences consist of non-coding regions called introns and protein-coding regions called exons. The designed primers for the MEF2A gene (GenBank accession number: XM_046924485.1) were positioned in exon 3 and exon 4, ensuring that the targeted DNA fragment corresponded to the protein-coding regions. Similarly, the primers for the ATF3 gene (GenBank accession number: XM_046939659.1) were positioned in exon 7 and exon 8. By strategically designing primers in the exon regions, the amplification process can selectively target the desired DNA fragments for further analysis (Table 1).

**Table 1:** Gene primer sequence

Name	Gene number	Strand	Sequence (5′-3′)	Size (bp)	Annealing temperature (°C)
MEF2A	XM_046924485.1	Forward	CAGCCAGCTCATCGTTAACAG	119	62
		Reverse	CATTCCACCAGCATTGCCAG		
ATF3	XM_046939659.1	Forward	CTTTGGATACGGTGACAGTG	134	62
		Reverse	GTTTCGGCACTTTGCAGC		
GAPDH	NM 204305.2	Forward	CTATCTTCCAGGAGCGTGAC	140	62
		Reverse	GCGTGTTATCATCTCAGCTC		

### Real-time PCR (RT-PCR)

RT-PCR was carried out using a reaction mixture that included 10 µl Toyobo ThunderBird, 8 µl nucleous-free water (NFW), 0.5 µl forward primer, and 0.5 µl reverse primer. Amplification was carried out with pre-denaturation at 95 °C for 5 min, denaturation at 95 °C for 10 s, annealing at 61 °C for 45 s, extension at 95 °C for 5 s, and final extension at 95 °C for 30 s. The amplification process on the RT-PCR machine lasts for 40 cycles. The results obtained were an amplification graph, melt curve graph, melt peak graph, and cycle quantification value. Gene expression values were displayed using the GraphPad Prism software version 9.

### Data analysis

The collected data on the weight of internal organs in crossbred chickens were organized and subjected to statistical analysis using analysis of variance (ANOVA) based on a completely randomized design (CRD). If significant differences were observed among the treatments, post hoc analysis was conducted using Duncan’s Multiple Range Test (DMRT) to determine the specific treatment effects. The mathematical model for a completely randomized design is represented as follows: *Yij* = *µ* + *α* + *εij*. Description: *Yij* = observation in the treatment; *µ* = general mean value; *α* = effect of the treatment; εij = experimental error in the treatment; *i* = 1, 2, 3; *j* = 1, 2, 3, 4, 5.

The quantification values of the MEF2A gene and ATF3 gene expression were calculated using the formula 2^−∆∆Ct^ which is used to compare the CT value of the target gene with GAPDH [36]. The difference between CT of the target gene and CT of the control gene (GAPDH) is ΔCT, while the difference between ΔCT of the target gene and ΔCT of the GAPDH gene is ΔΔCT [37]. The selection of the control sample is based on the treatment sample with the lowest protein level. The results of the difference between the target gene and the control gene (fold change) continued using analysis of variance (ANOVA).

## Result

### Weight of internal organs in crossbred local chickens

Based on the results of the study in Table 2, Table 2 presents the results indicating the significant effects (*P* < 0.05) of different protein levels on the heart weight of local crossbred chickens. Treatment 3 exhibited the highest heart weight, measuring 5.18 ± 0.56 g. This result differed by 0.95 g from treatment 2 and by 0.66 g from treatment 1. Furthermore, statistical analysis revealed a highly significant effect (*P* < 0.01) of the treatment on the pancreas weight. Treatment 2 yielded the highest pancreas weight of 3.04 ± 0.56 g, with a difference of 0.57 g from treatment 1 and 0.95 g from treatment 3. However, the treatment did not demonstrate a significant effect (*P* > 0.05) on the gizzard weight and spleen weight. Treatment 1 exhibited the highest gizzard weight at 27.64 ± 4.64 g, differing by 0.22 g from treatment 3 and by 0.75 g from treatment 2. Treatment 2 resulted in the highest spleen weight, measuring 2.80 ± 1.22 g, with a difference of 0.32 g from treatment 1 and 0.89 g from treatment 3.

**Table 2:** Effect of different protein level treatments on internal organ weight of crossbred local chicken

Variable	Treatment		
	T1	T2	T3
Heart weight (g)	4.52 ± 0.48^ab^	4.23 ± 0.60^a^	5.18 ± 0.56^b^
Gizzard weight (g)	27.64 ± 4.64	26.89 ± 4.16	27.42 ± 3.62
Pancreas weight (g)	2.47 ± 0.32^ab^	3.04 ± 0.56^b^	2.09 ± 0.15^a^
Spleen weight (g)	2.48 ± 1.05	2.80 ± 1.22	1.91 ± 0.40

^a-b^Different lowercase superscript notations in the same row indicate a highly significant effect (*P* < 0.01)

### Expression of MEF2A gene and ATF3 gene

**Fig. 1:**
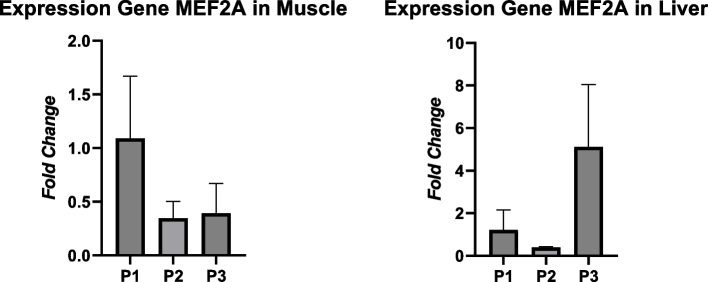
Expression gene MEF2A in the muscle tissue and liver tissue

**Fig. 2:**
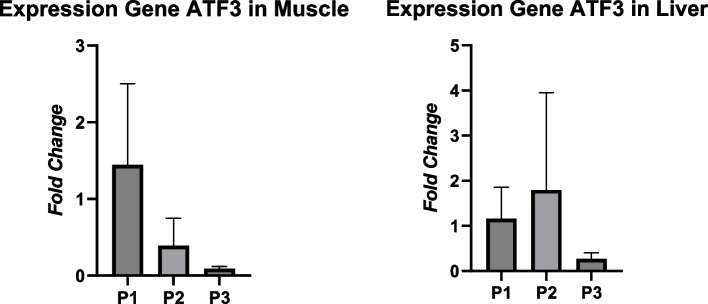
Expression gene ATF3 in the muscle tissue and liver tissue

The RT-PCR analysis revealed the expression of the MEF2A gene, indicating upregulation in both muscle tissue and liver tissue. However, the different protein treatments did not exhibit a significant difference (*P* > 0.05) in the MEF2A gene expression in muscle tissue, with the highest expression value recorded as 1.09 ± 0.58. On the other hand, the treatment had a significant effect (*P* < 0.05) on the MEF2A gene expression in liver tissue, with the highest expression value observed as 5.12 ± 2.93. Moreover, the treatment of different protein levels did not result in a significant difference (*P* > 0.05) in the ATF3 gene expression in both muscle tissue and liver tissue. Treatment 1 displayed high expression of the ATF3 gene in muscle tissue, measuring 1.44 ± 1.06, while treatment 2 exhibited high expression of the ATF3 gene in liver tissue, measuring 1.79 ± 2.15 (Figs. 1 and 2).

## Discussion

Heart weight is a multifactorial trait influenced by various factors such as breed, age, activity, and environment [17]. The weight of the heart is closely associated with blood flow, where a higher heart weight indicates smoother blood flow, thereby affecting the metabolic processes in the chicken’s body [35]. The T3 treatment with feed containing protein of 22% for the starter and 20% for the finisher showed the highest result of 5.18 g. Feed substances including digested protein enter the blood circulation through the capillaries of the small intestine wall which then collect in the portal vein. Blood containing protein after entering the liver then goes to the heart through the intermediary of the hepatic vein. One of the feed ingredients used in this research that has a significant difference between treatments is soybean meal. The content of soybean meal is a variety of amino acids and some anti-nutritional substances such as oxalic acid and trypsin inhibitors. Chickens will respond to feeds that contain antinutrients as exogenous which can interfere with the absorption and metabolic processes of nutrients in the chicken body [18]. The heart is one part of the internal organ that a function as a circulation system that functions to transfer blood from the heart organ to the whole body then the blood is returned to the heart, the existence of this system makes the heart organ very vulnerable to toxins and food substances in the ration. The secretion of trypsin which functions to digest protein will increase due to the enlargement of the pancreas. The protein content of 20% for starter and 18% for finisher influenced the weight of the pancreas 3.04 g in crossbred local chickens. The use of protein-containing synthesized amino acids in the feed will have an impact on the enlargement of the pancreas [19]. The increase in pancreas weight is one form of adaptation as a fulfillment of the increasing need for digestive enzymes [20]. Enzymatic processes in poultry occur in the duodenum, jejunum, and ileum with the help of amylase, trypsin, and lipase enzymes produced by the pancreas. High enzyme activity in the pancreas and intestine will increase chicken growth [21].

The use of feed with different protein levels has a significant effect on gizzard weight and spleen weight. Feeding that is not by the needs of livestock in terms of quantity and quality will cause the thickening of the meat veins so that the weight of the gizzard increases [4]. One of the feed ingredients whose composition is significantly different in each treatment is the bran separator. Bran is a by-product of the agricultural sector in the form of the outermost cuticle layer of rice which contains high crude fiber [22]. The use of feed ingredients that contain high crude fiber will increase the weight of the internal organs of chickens that work mechanically, one of which is the gizzard. The high content of crude fiber in the feed ration will increase the work of the gizzard so that the weight of the gizzard organ increases [17]. T1 with the highest crude fiber content of all treatments of 9.18% for the starter and 7.42 for the finisher influenced the highest gizzard weight of 27.64 g. The spleen is a lymphoid organ as a place of maturation and formation of antibody cells to protect against toxic attacks from consumed feed [23]. These foreign objects that enter the spleen will cause a macroscopic reactive process so that the spleen occurs swelling. Amino acid stimulation which is the target of protein synthesis in the liver, pancreas, and spleen will work as a mediator in metabolic pathways to support the body’s protein synthesis process [20].

The fold change value is a crucial parameter used to interpret the expression of target genes, indicating whether they are upregulated or downregulated. Treatment 1, with a protein content of 18% in the starter phase and 16% in the finisher phase, exhibited the highest gene expression with a value of 1.09 ± 0.58 compared to the other treatments. These findings suggest that the MEF2A gene, when subjected to lower protein treatments, can exhibit increased expression in muscle tissue. Interestingly, increased protein intake does not consistently result in elevated MEF2A gene expression in muscle tissue. This could be attributed to the MEF2A gene’s low sensitivity to protein variations in the muscle tissue of local crossbred chickens. Pro-myogenic genes such as IGF1, MRF4, MDF1, and MEF2A are expressed at higher levels in broilers characterized by rapid growth [24]. Rapid growth is not solely determined by a high-protein diet but rather by meeting the body’s protein requirements. The mRNA transcription level of the MEF2A gene reflects its role in controlling skeletal muscle regeneration and metabolic processes [25]. The muscle tissue samples of this study used the pectoral muscle (pectoralis major) which is thought to affect the level of MEF2A gene expression. This is in accordance with the opinion of [26] that the MEF2A gene in muscle tissue is involved in various responses including skeletal muscle myofiber differentiation, cardiac muscle development, and smooth muscle growth. Organ or tissue samples used can affect the MEF2A gene expression [12].

The results of this study showed that treatment 3 with 22% protein for the starter and 20% for the finisher gave MEF2A gene expression of 0.39 ± 0.28. This result is the lowest expression result compared to other treatments in muscle tissue. The protein content given to local crossbred chicken is not fully used to fulfill growth needs, but also to fulfill other metabolic needs. Amino acids contained in protein are involved in metabolic processes, thus affecting growth, health, immunity, and body protein accumulation [27].

MEF2A gene expression value in liver tissue is higher than the expression in muscle tissue. The results showed the MEF2A gene expression of treatment 3 in liver tissue was 5.12 ± 2.93. In contrast to the opinion of [12] that the level of MEF2A gene expression in chickens is highly expressed in muscle, heart, and brain tissues, while low expression in kidney and liver tissue. The high expression of the MEF2A gene in liver tissue is due to the amount of feed protein absorbed by the intestine directly into the liver, so the amount of protein has not been reduced for other metabolic processes. Protein metabolism in the liver starts from the process of protein hydrolysis in the intestine into peptides and free amino acids with the help of proteases and peptidases. The amino acids are absorbed by the erythrocytes and forwarded to the portal vein. The amino acids then enter the liver and spread to other tissues through systemic circulation [5]. The high level of protein in the liver is also due to the liver’s function as a site of phospholipid and cholesterol synthesis. The process requires proteins to bind with the lipids, thus forming lipoproteins. The transport of triglycerides from the liver to the adipose tissue is assisted by various classes of lipoprotein particles. The classification of lipoproteins is based on the proportion of proteins and lipids. The classes of lipoprotein particles that undergo synthesis and secretion in the liver are very low-density lipoproteins (VLDLs), intermediate-density lipoproteins (IDLs), low-density lipoproteins (LDLs), and high-density lipoproteins (HDLs) [28].

The ATF3 gene is a stress-induced transcription factor that plays a role in modulating metabolic processes, immunity, and oncogenesis [13]. Treatment 1 with a protein content of 18% for the starter and 16% for the finisher gave the highest results on the ATF3 gene expression in muscle tissue. Muscle tissue has a high nitrogen content in the body, so it is more sensitive to steroid hormones which cause a decrease in growth, muscle mass, and stress [29]. Increased stress adversely affects growth and health in poultry, due to decreased immunity and excess fat accumulation [14]. ATF3 gene expression accumulates rapidly in response to serum, tissue growth, and fibroblast growth factors after hepatectomy of the liver during the regeneration process [30]. One of the contributing factors is that stress levels increase when chickens are fed feed that does not meet their needs. Protein contains amino acids that play a role in muscle growth, membrane glycoproteins, and as precursors of DNA/RNA synthesis [38]. High protein content in feed affects the optimal work of metabolism [31]. Metabolism that does not run normally will interfere with the process of development and growth of chickens.

Treatment 2 with a protein content of 20% for the starter and 18% for the finisher gave the highest ATF3 gene expression of 1.79 ± 2.15 in liver tissue. This result is thought to be due to protein content that is not in accordance with the needs of local crossbred chicken, resulting in increased stress levels. The ATF3 gene has a negative function regulating the expression of adiponectin genes in adipocyte cells and adiponectin receptors in insulin-sensitive liver cells [32]. The liver in metabolic and homeostatic processes acts as a producer of body biochemicals and is responsible for synthesis, excretion, detoxification, and metabolism. The lack of nutrients including protein in the liver has an impact on lipid and protein metabolism disorders in the liver [5]. Treatment 2 showed that the ATF3 gene was highly expressed and positively correlated with a pancreas organ weight of 3.04 g and spleen weight of 2.8 g. The increase in the weight of the pancreas and spleen organs is thought to be low in antibacterial genes, so bacteria develop rapidly and interfere with the work of the pancreas and spleen organs. ATF3 gene expression correlates with antibacterial gene levels. Cells or tissues that have low ATF3 gene expression have low levels of antibacterial genes [33].

Treatment 3 with a protein content of 22% for the starter and 20% for the finisher showed the lowest ATF3 gene expression of 0.27 ± 0.13 in liver tissue. This result was positively correlated with the weight of visceral organs, namely the pancreas and spleen, which showed the lowest weight. This result is thought to be due to the provision of 22% protein for the starter and 20% for the finisher in accordance with the needs of local crossbred chickens. The stress level received by the chickens was low. High stress levels in chickens affect amino acid metabolism and increase the process of liver gluconeogenesis [34]. The protein excess received by the liver will be converted into ammonia and then disposed of. The liver’s role is to catabolize amino acids from excess protein and those that are not used for synthesis in tissues or hormones [5]. Protein in poultry tissues regenerates along with the release of endogenous amino acids. A balanced protein requirement will increase metabolism in the liver. There are many metabolic reactions that convert metabolites into essential amino acids [14]. Liver hepatocytes also play a role in triglyceride storage, lipoprotein synthesis, phospholipid synthesis, fatty acid metabolism into ATP, and energy release to tissues [5].

## Conclusion

In conclusion, this research examined the impact of different protein levels in the diet on internal organ weight and the expression of MEF2A and ATF3 genes in crossbred local chickens. The findings indicate that feeding crossbred chickens with diets containing crude protein levels ranging from 18 to 22% for the starter phase and 20 to 22% for the finisher phase resulted in normal heart, pancreas, and spleen weights. However, abnormal gizzard size was observed. Interestingly, increased protein utilization did not lead to an increase in MEF2A gene expression in muscle and liver tissues. Notably, the MEF2A gene exhibited high expression in muscle tissue when fed with a protein content of 18% for the starter phase and 16% for the finisher phase. Conversely, the MEF2A gene showed high expression in liver tissue when fed with a protein content of 22% for the starter phase and 20% for the finisher phase. Furthermore, ATF3 gene expression in muscle tissue exhibited a negative correlation with increasing protein content in the diet. However, the ATF3 gene was highly expressed in liver tissue when fed with a protein content of 20% for the starter phase and 18% for the finisher phase. These findings demonstrate that the MEF2A and ATF3 genes display distinct patterns of gene expression in different tissues.

## Data Availability

All data used are primary data from research results and research materials using tools belonging to the biotechnology laboratory of the Faculty of Animal Science Universitas Brawijaya.

## Abbreviations

MEF2A: Myogenic enhancer transcription factor 2A
ATF3: Activating transcription factor 3
GAPDH: Glyceraldehyde 3-phosphate dehydrogenase
RT-PCR: Real-time polymerase chain reaction
bp: Base pair
RNA: Ribonucleic acid
DNA: Deoxyribonucleic acid
EDTA: Ethylenediaminetetraacetic acid
